# Impact of Comorbidities on SARS-CoV-2 Viral Entry-Related Genes

**DOI:** 10.3390/jpm10040146

**Published:** 2020-09-25

**Authors:** Joshua D. Breidenbach, Prabhatchandra Dube, Subhanwita Ghosh, Belal N. Abdullah, Nikolai N. Modyanov, Deepak Malhotra, Lance D. Dworkin, Steven T. Haller, David J. Kennedy

**Affiliations:** 1 Department of Medicine, The University of Toledo College of Medicine and Life Sciences, Toledo, OH 43614, USA; joshua.breidenbach@rockets.utoledo.edu (J.D.B.); prabhatchandra.dube@utoledo.edu (P.D.); subha.ghosh1154@gmail.com (S.G.); belal.abdullah@rockets.utoledo.edu (B.N.A.); deepak.malhotra@utoledo.edu (D.M.); lance.dworkin@utoledo.edu (L.D.D.); 2 Department of Physiology and Pharmacology, The University of Toledo College of Medicine and Life Sciences, Toledo, OH 43614, USA; nikolai.modyanov@utoledo.edu

**Keywords:** SARS-CoV-2, COVID-19, comorbidities, tropism, renin-angiotensin aldosterone system, bradykinin, transmembrane serine proteases, heparan sulfate proteoglycans

## Abstract

Viral entry mechanisms for severe acute respiratory syndrome coronavirus 2 (SARS-CoV-2) are an important aspect of virulence. Proposed mechanisms involve host cell membrane-bound angiotensin-converting enzyme 2 (ACE2), type II transmembrane serine proteases (TTSPs), such as transmembrane serine protease isoform 2 (TMPRSS2), lysosomal endopeptidase Cathepsin L (CTSL), subtilisin-like proprotein peptidase furin (FURIN), and even potentially membrane bound heparan sulfate proteoglycans. The distribution and expression of many of these genes across cell types representing multiple organ systems in healthy individuals has recently been demonstrated. However, comorbidities such as diabetes and cardiovascular disease are highly prevalent in patients with Coronavirus Disease 2019 (COVID-19) and are associated with worse outcomes. Whether these conditions contribute directly to SARS-CoV-2 virulence remains unclear. Here, we show that the expression levels of ACE2, TMPRSS2 and other viral entry-related genes, as well as potential downstream effector genes such as bradykinin receptors, are modulated in the target organs of select disease states. In tissues, such as the heart, which normally express ACE2 but minimal TMPRSS2, we found that TMPRSS2 as well as other TTSPs are elevated in individuals with comorbidities compared to healthy individuals. Additionally, we found the increased expression of viral entry-related genes in the settings of hypertension, cancer, or smoking across target organ systems. Our results demonstrate that common comorbidities may contribute directly to SARS-CoV-2 virulence and we suggest new therapeutic targets to improve outcomes in vulnerable patient populations.

## 1. Introduction

Comorbidities such as diabetes, chronic lung disease, and cardiovascular disease are highly prevalent in patients with COVID-19 and are associated with worse outcomes [1,2]. However, whether these conditions contribute directly to SARS-CoV-2 virulence or simply worsen outcomes through independent mechanisms and reflect the general disease burden of the population remain unclear [2,3]. Furthermore, clinical and experimental evidence has demonstrated that, in addition to the lungs, SARS-CoV-2 infection of other target organ systems such as the heart, kidney, and blood may have deleterious consequences that can potentially compromise organ function and compound disease burden in COVID-19 patients [4,5].

The spike (S) protein of SARS-CoV and SARS-CoV-2 is a key facilitator for host cell entry through its binding to host cell membrane-bound ACE2 [6]. Therefore, the impact of the modulation of *ACE2* and the related renin-angiotensin-aldosterone system genes on COVID-19 has been an area of interest [3], and these genes were included in the current study. Heparan sulfate chains on heparan sulfate proteoglycans (HSPGs) have been reported to bind the spike protein of SARS-CoV-2 [7,8]. In addition to the widely reported ACE2, host cell membrane bound HSPGs, such as syndecans 1-4 (SDC1-4), glycosylphosphatidylinositol-anchored proteoglycans (glypicans 1-6 (GPC1-6)), betaglycan (TGFBR3), neuropilin-1 (NRP1) and CD44 may serve as alternative or complementary binding molecules [9]. Furthermore, downstream effector molecules such as those involved in the bradykinin system have important intersections with the renin-angiotensin-aldosterone system and its metabolites and have been suggested as potential drivers of a “Bradykinin Storm” which may drive adverse outcomes in COVID-19 patients [10].

After binding, cleavage of the S protein is necessary for S protein-mediated membrane fusion which drives viral entry into host cells. This proteolytic activity may be cathepsin-L dependent and occur upon pH change in cellular endosomes, or it may occur through the action of membrane bound serine proteases at the host cell membrane surface or within vesicles [11,12]. Additionally, viral entry mechanisms have been proposed which involve the cleavage of ACE2 by membrane bound serine proteases, leading to increased viral entry [11]. In fact, the importance of serine proteases in a viral entry mechanism may be emphasized by the success of serine protease inhibition in vitro [6]. However, while this and current mechanistic studies have focused on the proteolytic activity of TMPRSS2 and human airway trypsin-like protease (HAT, also referred to as TMPRSS11D), additional TTSPs are hypothesized to have similar extracellular cleavage activity [13], and were included in the current study. Unlike in SARS-CoV, the S protein of SARS-CoV-2 contains a multibasic furin cleavage site and recent evidence suggests this activity may prime the newly formed virion for membrane fusion, even before escape. This represents either a membrane bound protease independent mechanism, or the existence of a two-step process as is the case for the Middle East respiratory syndrome (MERS) [8,14,15]. Importantly, the influence of comorbidities on these genes is unknown. Thus, in the current study we examined the influence of comorbidities on the expression of key renin-angiotensin-aldosterone system and protease genes which may prime the cell entry mechanisms for SARS-CoV-2 across various organ systems.

## 2. Materials and Methods

**Figure 1:**
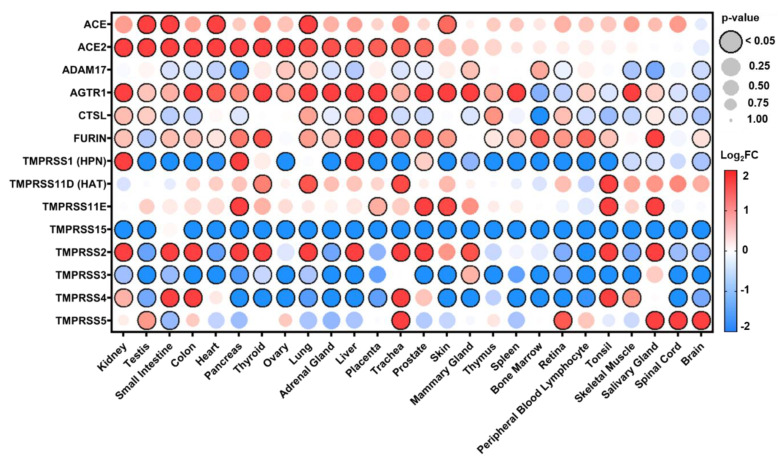
SARS-CoV-2 viral-entry gene expression across healthy human tissues. Expression of select genes related to SARS-CoV-2 viral-entry in healthy volunteers across 26 different body sites. Expression is displayed as logarithm to base 2 of the fold change (log_2_FC) relative to Universal Human Reference RNA as described in the GEO DataSet (GDS3113), *n* = 3. Heatmap coloration is set to a scale of −2 (blue) to 2 (red) and values beyond this range are shown as either −2 or 2, respectively. Statistical significance is represented proportional to dot size where largest dot size indicates highest statistical significance as indicated in the key and a black border designates *p* < 0.05.

In the current study, we examined the expression levels of SARS-CoV-2 entry-related genes in target organ systems including pulmonary, renal, cardiac, and peripheral blood mononuclear cells (PBMCs) from 1968 patients across a variety of common comorbidities including hypertension (*n* = 94 hypertensives vs. *n* = 61 normotensives), diabetes (*n* = 131 diabetic vs. *n* = 101 normoglycemic), obesity (*n* = 56 obese vs. *n* = 58 healthy weight), chronic lung disease (*n* = 200 asthmatic vs. *n* = 106 non-asthmatic and Chronic Obstructive Pulmonary Disease (COPD) *n* = 94 vs. healthy tissue *n* = 42) and cardiovascular disease (ischemic *n* = 23 and dilated *n* = 54 cardiomyopathy vs. healthy tissue *n* = 55), as well as other common pathologies such as chronic kidney disease (CKD) and cancer. Differential gene expression was curated from genetic data deposited in the National Center for Biotechnology Information (NCBI), U.S. National Library of Medicine, Gene Expression Omnibus (GEO) DataSets and the European Molecular Biology Laboratory (EMBL), European Bioinformatics Institute (EBI). Exhaustive queries in these databases in the form of “[tissue] and [hypertension, diabetes, obesity, smoking, asthma]” were performed using the NCBI and iLINCS website (ilincs.org) and all hits were considered. DataSeries not containing disease state vs. healthy controls for the tissues concerned were excluded. Differential Expression analysis was performed using the GEO2R (NCBI) interactive web tool, and the iLINCS integrative web platform for the analysis of the Library of Integrated Network-Based Cellular Signatures (LINCS). The expression values in healthy tissues reported in Figure 1 and Figure S1 are reported as logarithm to base 2 of the fold change (log_2_FC) relative to Universal Human Reference RNA as described in the GEO DataSet (GDS3113). For comorbidity disease condition data, expression levels are described in log_2_FC in relation to each unaffected control group specific to each DataSeries. The dot plots were generated using R programming language and ggplot2 with heatmap-style coloration indicating the log_2_FC [16]. Statistical analysis was performed by the GEO2R web tool or iLINCS web platform and the dot sizes are proportional to the *p*-value where largest dot sizes indicate highest statistical significance and a black border indicates *p* < 0.05 [17]. All log_2_FC values outside of the −2 to 2 range are shown as either −2 or 2. DataSeries in this analysis are linked in each figure to Appendix A and described in Supplementary File 1.

## 3. Results

### 3.1. Distribution of Expression in Healthy Tissues

To better understand the expression patterns of TTSPs and other viral entry-related genes in a healthy setting, expression data were analyzed for 26 different body sites from 3 healthy individuals (Figure 1 and Figure S1). In healthy human tissues, there was a diverse transcription of renin-angiotensin-aldosterone system related genes (*ACE*, *ACE2*, and *AGTR1* (Figure 1) and *BDKBR1* and *BDKBR2* (Figure S1)) as well as proteases (*ADAM17*, *CTSL*, *FURIN*, *TMPRSS1*-*5*, *TMPRSSD*, *TMPRSSE*, and *TMPRSS15*), and heparan sulfate proteoglycans (*CD44*, *GPC1*, *GPC3-6*, *NRP1*, *SDC1*, *SDC2*, *SDC4*, and *TGFBR3* (Figure S1)) which may prime the cell entry mechanisms for SARS-CoV-2 across various organ systems. The results from our analysis are in agreement with previously reported expression patterns [18]. Specifically, both surveys found high levels of *ACE2* and/or *TMPRSS2* expression in the kidney, colon, heart, lung, and prostate. In addition, our analysis suggests small intestine, pancreas, thyroid, liver, trachea, and prostate as sites of high expression. 

### 3.2. Expression in Common Comorbidities

Additionally, we examined the impact of a variety of common comorbidities on the expression of these genes in select organ systems (pulmonary, renal, cardiac, and blood tissues) based on their relevance to the infection and expression levels at baseline. Expression data displayed in each comorbidity dot plot is displayed from greatest (left) to least (right) expression of *ACE2*.

#### 3.2.1. Expression in Pulmonary Tissues in Comorbid States

**Figure 2:**
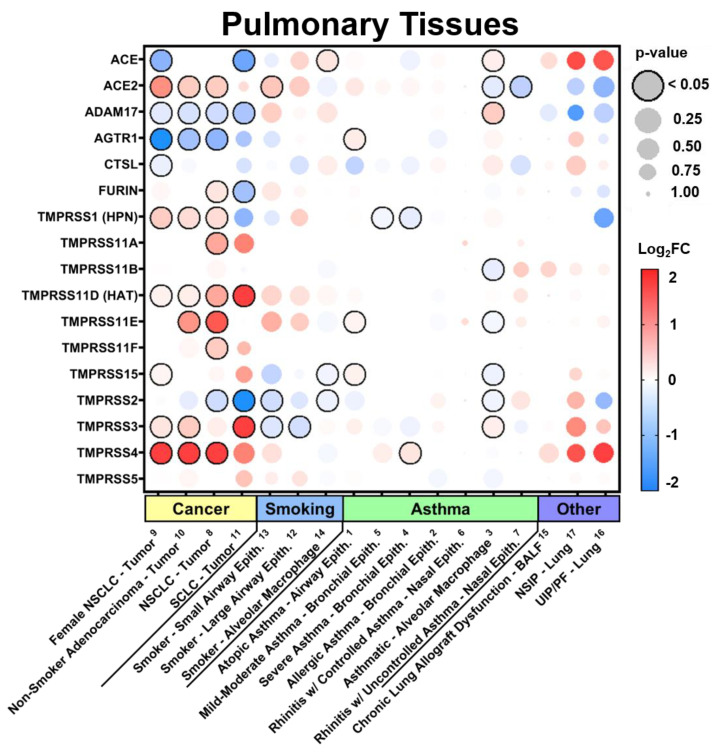
SARS-CoV-2 viral-entry gene expression in pulmonary tissues across comorbidities. Expression of select SARS-CoV-2 viral-entry genes in pulmonary tissues across 17 DataSets representing various common comorbidities. Values are displayed as logarithm to base 2 of the fold change (log_2_FC) in comparison with respective unaffected control samples from each DataSet. Comorbidity groups and each DataSet within are sorted from greatest (**left**) to least (**right**) expression of *ACE2*. Heatmap coloration is set to a scale of −2 (blue) to 2 (red) and values beyond this range are shown as either −2 or 2, respectively. Statistical significance is represented proportional to dot size where largest dot size indicates highest statistical significance as indicated in the key and a black border designates *p* < 0.05. Superscript numbers link each DataSet to additional information found in Appendix A and Supplementary File 1. BALF = Bronchoalveolar lavage fluid; NSCLC = non-small cell lung carcinoma; NSIP = non-specific interstitial pneumonia; SCLC = small cell lung carcinoma; UIP/PF = usual interstitial pneumonia/idiopathic pulmonary fibrosis.

In pulmonary tissues (Figure 2 and Figure S2), we found the greatest increase in *ACE2* in cancer with substantial increases in nearly all of the TTSPs, which is consistent with their role in tumor cell proliferation, motility, and invasion [13]. While carcinomas resulted in variable changes to the HSPGs, TGFBR3 was notably decreased. Additionally, samples from patients with a history of smoking showed increases in *ACE2* in both small and large airways, consistent with recent findings [19]. While expression levels in the context of pre-existing asthma appeared to be largely unaffected in pulmonary tissues on average, there were more pronounced increases in bronchial compared to nasal epithelium. In nearly all comorbid DataSets, the HSPGs CD44 and NRP1 were consistently increased while bradykinin receptors were only increased in the setting of both Small cell and Non-small cell carcinoma (SCLC/NSCLC), Non-specific interstitial pneumonia (NSIP), and Usual interstitial pneumonia/Idiopathic pulmonary fibrosis (UIP/PF, Figure S2).

#### 3.2.2. Expression in Renal Tissues in Comorbid States

**Figure 3:**
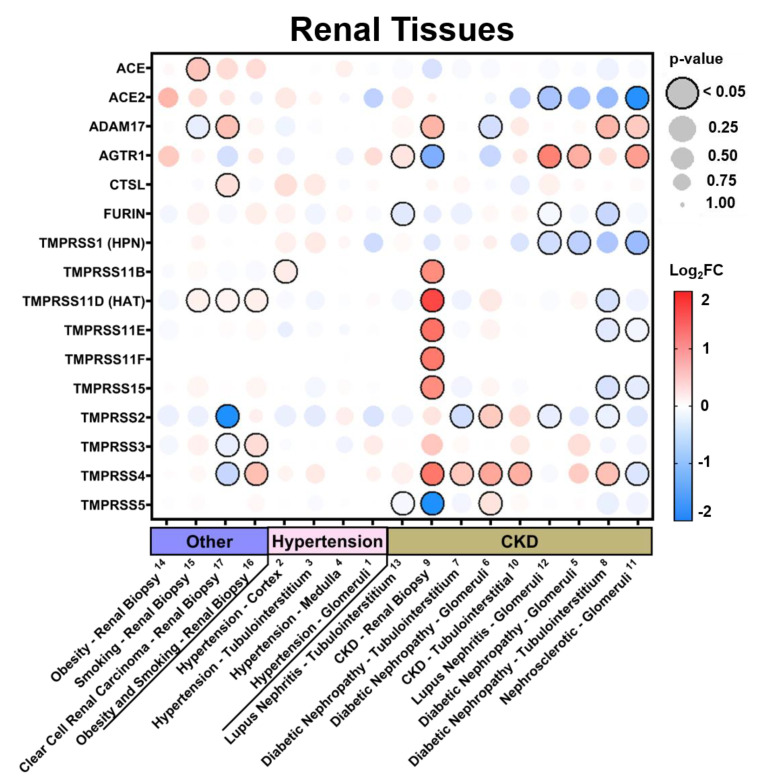
SARS-CoV-2 viral-entry gene expression in renal tissues across comorbidities. Expression of select SARS-CoV-2 viral-entry genes in renal tissues across 17 DataSets representing various common comorbidities. Values are displayed as logarithm to base 2 of the fold change (log_2_FC) in comparison with respective unaffected control samples from each DataSet. Comorbidity groups and each DataSet within are sorted from greatest (**left**) to least (**right**) expression of ACE2. Heatmap coloration is set to a scale of −2 (blue) to 2 (red) and values beyond this range are shown as either −2 or 2, respectively. Statistical significance is represented proportional to dot size where largest dot size indicates highest statistical significance as indicated in the key and a black border designates *p* < 0.05. Superscript numbers link each DataSet to additional information found in Appendix A and Supplementary File 1. CKD = chronic kidney disease.

In renal tissues (Figure 3 and Figure S3), we found the greatest expression of *ACE2* in obesity. Similar to what has been seen in pulmonary tissues, a history of smoking or cancer associated with an increase in *ACE2* as well as slight increases in TTSPs and HSPGs in renal biopsy. Hypertensives had increases in *ACE2*, *TMPRSS1*, and *TMPRSS4* in renal cortical and tubulointerstium, but not glomerular or medullar samples. Chronic kidney disease (CKD) resulted in the greatest diversity in the modulation of these genes, however, with consistent increases in *TMRPSS4* in 67% of DataSets from both tubular and glomerular origin. In nearly all comorbid DataSets, the HSPGs CD44 and NRP1 were consistently increased (Figure S3).

#### 3.2.3. Expression in Cardiac Tissues in Comorbid States

**Figure 4:**
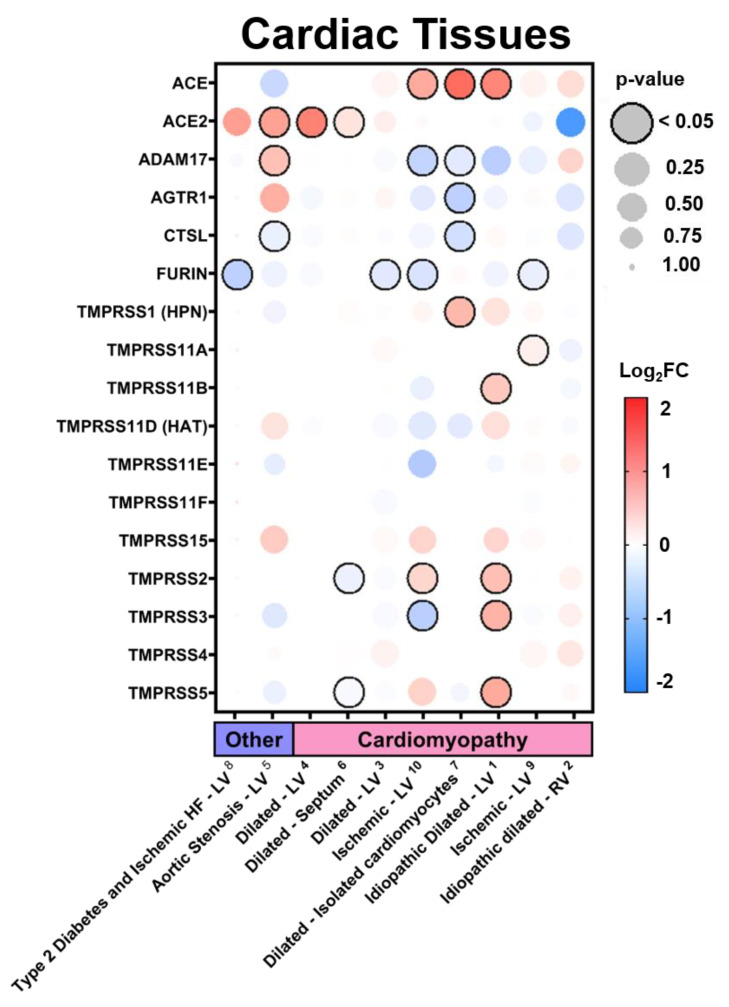
SARS-CoV-2 viral-entry gene expression in cardiac tissues across comorbidities. Expression of select SARS-CoV-2 viral-entry genes in cardiac tissues across 10 DataSets representing various common comorbidities. Values are displayed as logarithm to base 2 of the fold change (log_2_FC) in comparison with respective unaffected control samples from each DataSet. Comorbidity groups and each DataSet within are sorted from greatest (**left**) to least (**right**) expression of ACE2. Heatmap coloration is set to a scale of −2 (blue) to 2 (red) and values beyond this range are shown as either −2 or 2, respectively. Statistical significance is represented proportional to dot size where largest dot size indicates highest statistical significance as indicated in the key and a black border designates *p* < 0.05. Superscript numbers link each DataSet to additional information found in Appendix A and Supplementary File 1. HF = heart failure; LV **=** left ventricle; RV = right ventricle.

In cardiac tissues (Figure 4 and Figure S4), we found the greatest increases in *ACE2* in patients who had experienced heart failure with pre-existing diabetes and patients with aortic stenosis. While cardiomyopathies resulted in variable expression levels, increases in *ACE2* were found in left ventricle (LV) tissues while decreases were found in right ventricle (RV) tissues. On average, slight increases were found for many TTSPs with 33% of DataSets demonstrating increases in *TMPRSS2*. Consistent increases in the expression of HSPGs and bradykinin receptors were found in most of the diseased DataSets (Figure S4).

#### 3.2.4. Expression in Blood Tissues in Comorbid States

**Figure 5:**
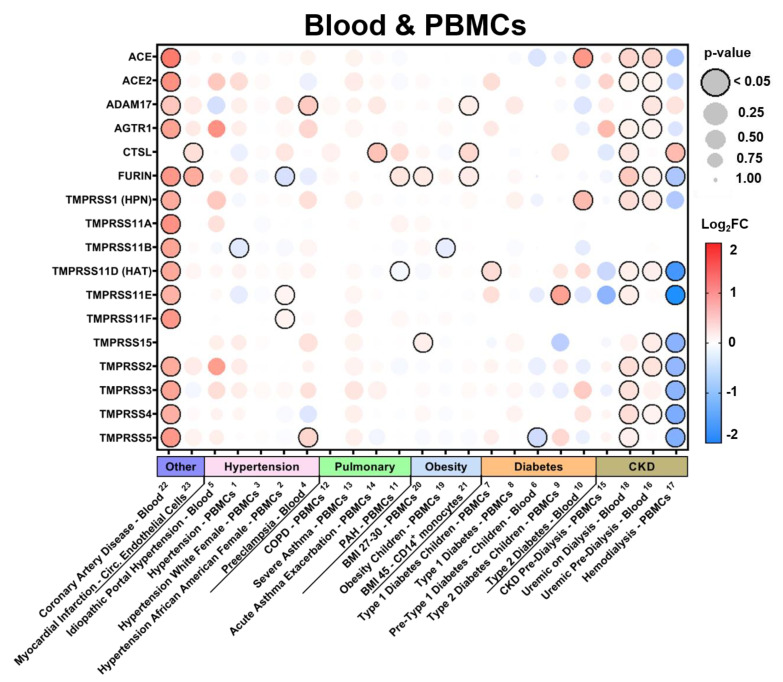
SARS-CoV-2 viral-entry gene expression in blood tissues across comorbidities. Expression of select SARS-CoV-2 viral-entry genes in blood tissues across 23 DataSets representing various common comorbidities. Values are displayed as logarithm to base 2 of the fold change (log_2_FC) in comparison with respective unaffected control samples from each DataSet. Comorbidity groups and each DataSet within are sorted from greatest (**left**) to least (**right**) expression of ACE2. Heatmap coloration is set to a scale of −2 (blue) to 2 (red) and values beyond this range are shown as either −2 or 2, respectively. Statistical significance is represented proportional to dot size where largest dot size indicates highest statistical significance as indicated in the key and a black border designates *p* < 0.05. Superscript numbers link each DataSet to additional information found in Appendix A and Supplementary File 1. BMI = body mass index; CKD = chronic kidney disease; COPD = chronic obstructive pulmonary disease; PBMCs = peripheral blood mononuclear cells; PAH = pulmonary arterial hypertension.

In blood (Figure 5 and Figure S5), remarkable increases in nearly all selected genes were found in patients with coronary artery disease. Hypertension and chronic lung pathologies resulted in slight, but consistent increases in most of the selected genes including *ACE2* and many of the TTSPs and HSPGs. Specifically, in hypertension, increases were found in at least 20% of DataSets for *ACE2*, *ADAM17*, *AGTR1*, *TMPRSS1*, *TMPRSS2*, *TMPRSS3*, *TMPRSS5*, *TMPRSS11A*, and *TMPRSS15*. In contrast to the results seen in renal tissues, obesity did not appear to modulate the expression of any of these genes except for SDC4 in circulating immune cells. Notably, increased expression levels were found in the context of type 1 diabetes, while mostly decreases were apparent in type 2 diabetes in whole blood and peripheral blood mononuclear cells (PBMCs). Lastly, highly variable modulation was found in the context of CKD with or without hemodialysis with increases in *ACE*, *ACE2*, *AGTR1*, *TMPRSS1*, *TMPRSS2*, *TMPRSS3*, *TMPRSS4*, *BDKRB1*, *BDKRB2*, and most of the HSPGs in at least 25% of the DataSets.

## 4. Discussion

Investigations since the SARS-CoV outbreak in 2002–2003 and especially more recently have focused on the expression of ACE2 and its relationship to viral entry [6]. In a recent thorough report, *ACE2* and *TMPRSS2* expression was mapped across various body sites in normal healthy tissue by single-cell RNA sequencing [18]. However, a more complete model of viral entry for SARS-CoV and SARS-CoV-2 describes a potential role for TMPRSS2, HAT, and possibly other TTSPs [11]. Because interest in TTSPs seems to have blossomed only within the last decade, their appearance on microarray technologies and therefore their appearance in these data are limited. However, understanding the modulation of the expression of these genes across various organ systems and in the context of common comorbidities should provide us with a more complete understanding of the potential impact of these comorbidities on viral proliferation. As demonstrated in our analysis, TTSPs are highly expressed in cancerous lung tissue and this is supported by recent literature concerning the involvement of these serine proteases in tumor cell proliferation, motility, and invasion [13].

Additional aspects to the mechanisms of viral entry for SARS-CoV-2 have been described. While ACE2 is heavily reported on, there is mounting evidence of HSPGs serving as additional binding molecules to hold the virus proximal to the host cell surface [7,8]. In addition, both cathepsin L and furin seem to be responsible for S protein cleavage in different contexts [12,14]. Therefore, understanding the modulation of viral entry during comorbid states will require an understanding of the expression of these genes as well.

Increases in the expression of viral entry-related genes as suggested by this data in common comorbidities across tissues such as hypertension, cancer, and a history of smoking may help to partially explain their association with higher morbidity and mortality in COVID-19. In other comorbidities, such as obesity and diabetes or in those with tissue specificity such as in cardiomyopathies and chronic lung disease, the lack of consistent alteration across tissues may suggest a mechanism for tropism of the virus. To be sure, while SARS-CoV and SARS-CoV-2 virulence is driven by factors outside of viral entry, it may be important to understand the influence of the varied expression of these genes among target organ systems and across comorbidities as demonstrated.

However, it is important to point out a few limitations to this analysis. While not the main focus of this study, the expression levels in healthy individuals were only based on a relatively limited sample size (*n* = 3). This is likely due to the cost of this analysis and the breadth of tissues analyzed for which it was chosen in this study. Regardless, this is meant to provide a starting reference point for the expression of these selected genes across various human tissues. In addition to comorbidities, as demonstrated in this study, gene expression is influenced by age, sex, race, geographical region, diet, etc., and these variables are not accounted for in this study due to the lack of uniformity in the existing metadata on each DataSet. However, details are available in the supplemental material (Supplementary File 1) and additional metadata are often available at the source database (NCBI-GEO, or EMBL-EBI) following the unique ID provided in Appendix A and Supplementary File 1. It should be stated that the expression of these genes in other tissues such as the vasculature and under different comorbid states may be important, as supported by the results of our analysis of whole blood in patients with coronary artery disease. While we did not find vascular DataSets with comparable expression data across similar comorbidities, we do note that the data in each of these analyses is derived from whole tissue homogenate and thus includes the vascular component of each tissue type. Furthermore, as SARS-CoV-2 mediated vascular complications related to vasodilation and vascular permeability have been noted, we included bradykinin receptors in the analysis as these have been suggested to participate in the vascular phenotype that accompanies COVID-19 [10]. Lastly, while gene expression was the focus of this analysis, this may not translate to differential protein abundance. As such, we do not suggest that changes in gene expression alone linearly influence viral entry or infection as the stoichiometry of these individual processes are not fully understood. However, multiple studies have confirmed the influence on viral infection by the knockout, inhibition, or overexpression of several of these genes and thus provide a rationale for examining their expression patterns in this setting [11,12].

In conclusion, the expression levels of SARS-CoV-2 viral-entry related genes in patients suffering from common comorbidities such as hypertension, cancer, a history of smoking, obesity, diabetes, cardiomyopathies, or chronic lung or kidney disease may be increased in target organ systems and be capable of directly contributing to infection. This represents an important step in designing effective therapeutic and preventative strategies to improve outcomes in vulnerable populations.

## Supplementary Materials

The following are available online at https://www.mdpi.com/2075-4426/10/4/146/s1, Figure S1: SARS-CoV-2 viral-entry gene expression across healthy human tissues, Figure S2: SARS-CoV-2 viral-entry gene expression in pulmonary tissues across comorbidities, Figure S3: SARS-CoV-2 viral-entry gene expression in renal tissues across comorbidities, Figure S4: SARS-CoV-2 viral-entry gene expression in cardiac tissues across comorbidities, Figure S5: SARS-CoV-2 viral-entry gene expression in blood tissues across comorbidities, Supplementary File 1.

## Appendix A

DataSeries in this analysis were as follows with GSEXXXX referring to NCBI-GEO and E-XXXX-XXXX referring to EMBL-EBI:

Pulmonary Tissues: (1, GSE18965; 2, GSE41649; 3, GSE2125; 4, E-GEOD-63142; 5, E-GEOD-63142; 6, E-GEOD-19187; 7, E-GEOD-19187; 8, E-GEOD-44077; 9, GSE19804; 10, E-GEOD-43458; 11, E-GEOD-60052; 12, GSE5057; 13, GSE3320; 14, GSE2125; 15, E-MTAB-6040; 16, GSE21411; 17, GSE21411);

Renal Tissues: (1, GSE104948; 2, GSE28345; 3, GSE104954; 4, GSE28360; 5, GSE104948; 6, GSE30528; 7, GSE104954; 8, GSE30529; 9, GSE66494; 10, GSE12682; 11, GSE20602; 12, GSE32591; 13, GSE32591; 14, GSE46699; 15, GSE46699; 16, GSE46699; 17, GSE46699);

Cardiac Tissues: (1, GSE1145; 2, GSE67492; 3, GSE42955; 4, GSE3585; 5, GSE10161; 6, GSE3586; 7, GSE120836; 8, GSE26887; 9, GSE42955; 10, GSE1145);

Blood and PBMCs: (1, GSE24752; 2, GSE75360; 3, GSE75360; 4, GSE48424; 5, GSE69601; 6, E-TABM-666; 7, GSE9006; 8, GSE55098; 9, GSE9006; 10, GSE23561; 11, GSE131793; 12, GSE42057; 13, GSE59019; 14, GSE16032; 15, GSE15072; 16, GSE37171; 17, GSE15072; 18, GSE37171; 19, GSE87493; 20, GSE69039; 21, GSE32575; 22, GSE23561; 23, GSE66360).
