# Supplementary material for: Impact of Comorbidities on SARS-CoV-2 Viral Entry-Related Genes
Source: J Pers Med. 2020 Sep 25;10(4):146. doi: 10.3390/jpm10040146 (PMC7720121; doi:10.3390/jpm10040146)
Supplement: Supplementary file 1 [file jpm-10-00146-s001.zip › Supplementary Figures Final proofread.docx]

Supplementary Figures


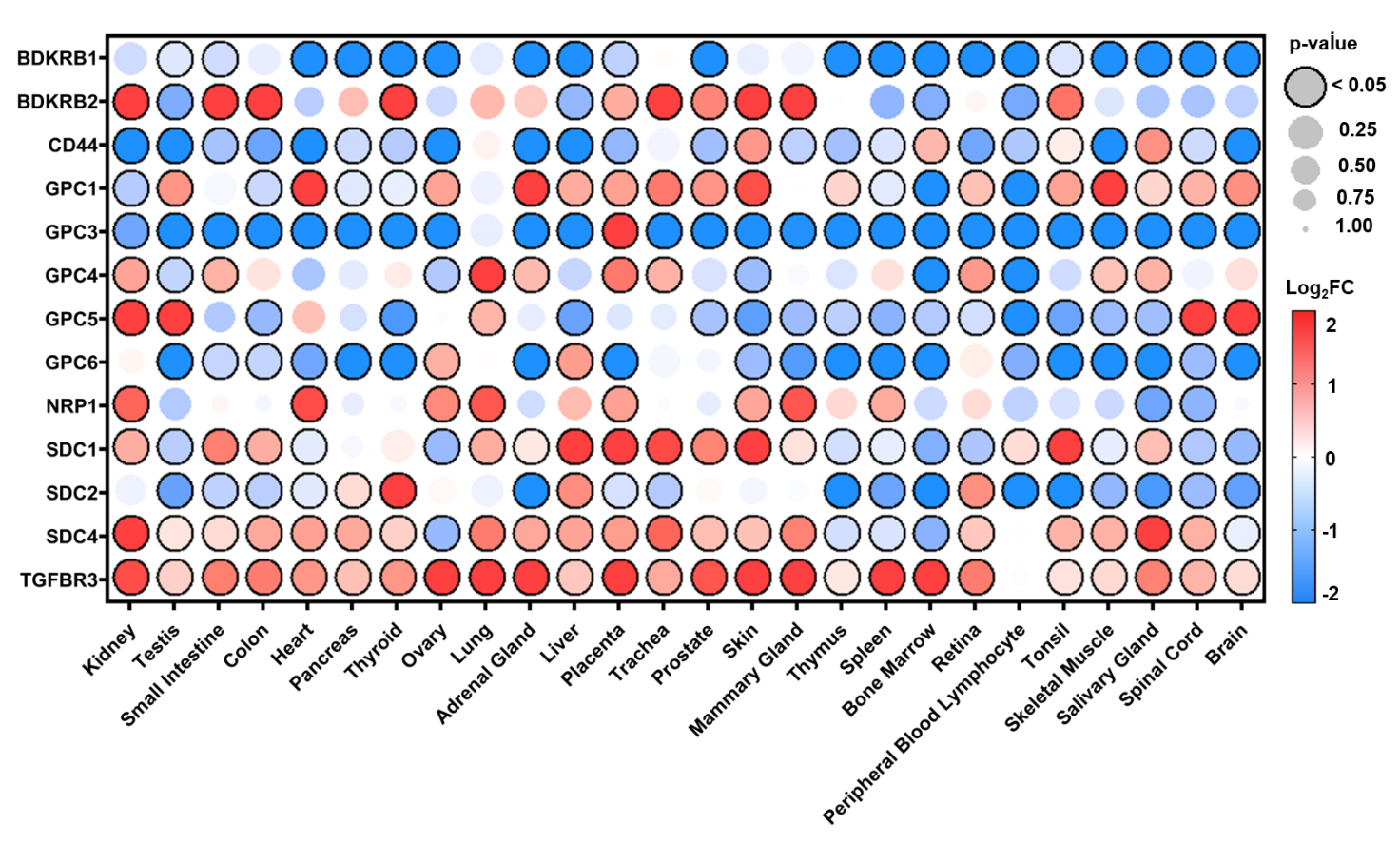


**Figure S1.** **SARS-CoV-2 viral-entry gene expression across healthy human tissues.** Expression of select genes related to SARS-CoV-2 viral-entry in healthy volunteers across 26 different body sites. Expression is displayed as logarithm to base 2 of the fold change (log_2_FC) relative to Universal Human Reference RNA as described in the GEO DataSet (GDS3113), n=3. Heatmap coloration is set to a scale of -2 (blue) to 2 (red) and values beyond this range are shown as either -2 or 2, respectively. Statistical significance is represented proportional to dot size where largest dot size indicates highest statistical significance as indicated in the key and a black border designates p < 0.05.


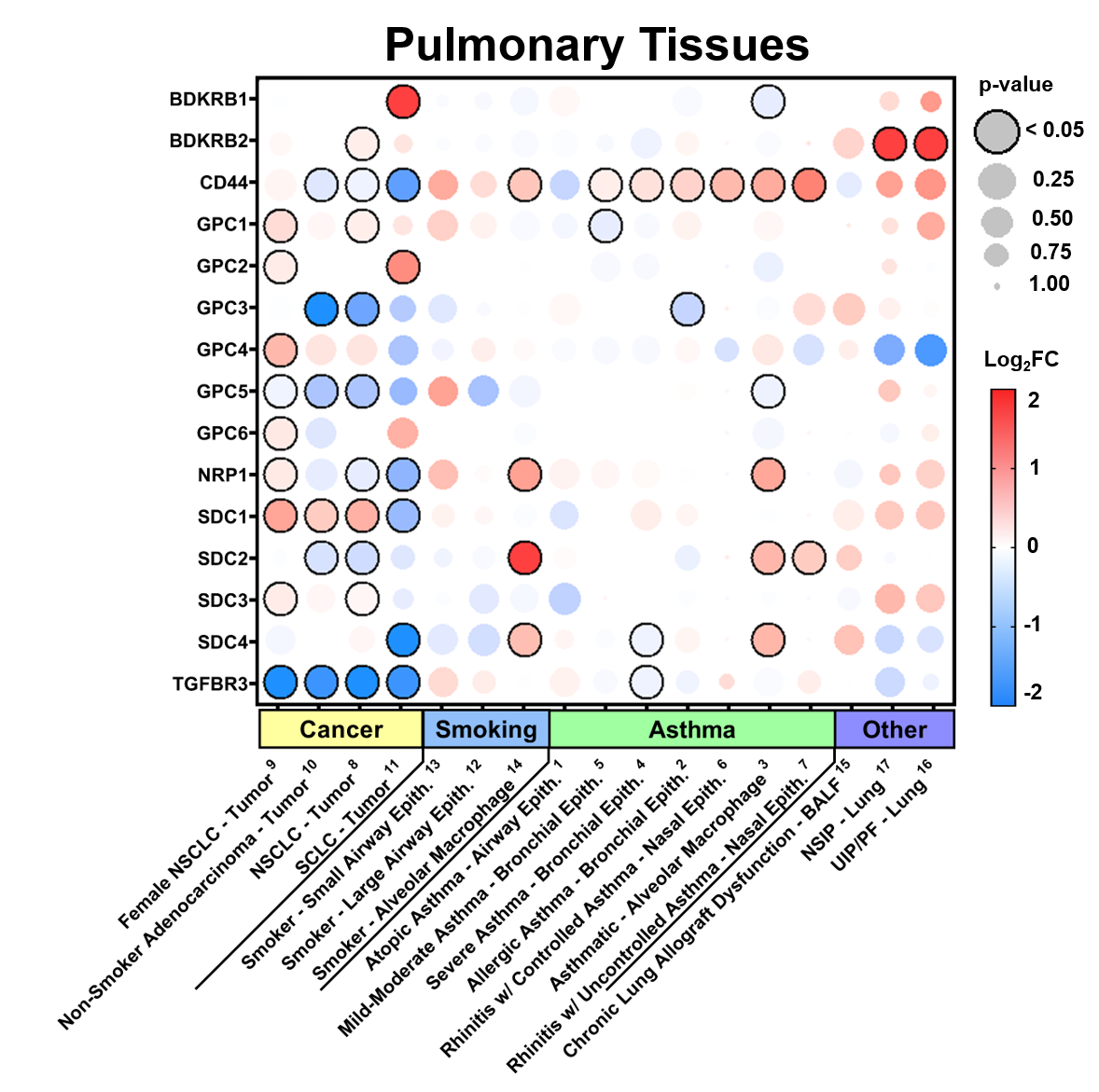


**Figure S2.** **SARS-CoV-2 viral-entry gene expression in pulmonary tissues across comorbidities.** Expression of select SARS-CoV-2 viral-entry genes in pulmonary tissues across 17 DataSets representing various common comorbidities. Values are displayed as logarithm to base 2 of the fold change (log_2_FC) in comparison with respective unaffected control samples from each DataSet. Comorbidity groups and each DataSet within are sorted from greatest (left) to least (right) expression of *ACE2*. Heatmap coloration is set to a scale of -2 (blue) to 2 (red) and values beyond this range are shown as either -2 or 2, respectively. Statistical significance is represented proportional to dot size where largest dot size indicates highest statistical significance as indicated in the key and a black border designates p < 0.05. Superscript numbers link each DataSet to additional information found in Appendix A and Supplementary File 1. BALF = Bronchoalveolar lavage fluid; NSCLC = non-small cell lung carcinoma; NSIP = non-specific interstitial pneumonia; SCLC = small cell lung carcinoma; UIP/PF = usual interstitial pneumonia/idiopathic pulmonary fibrosis.


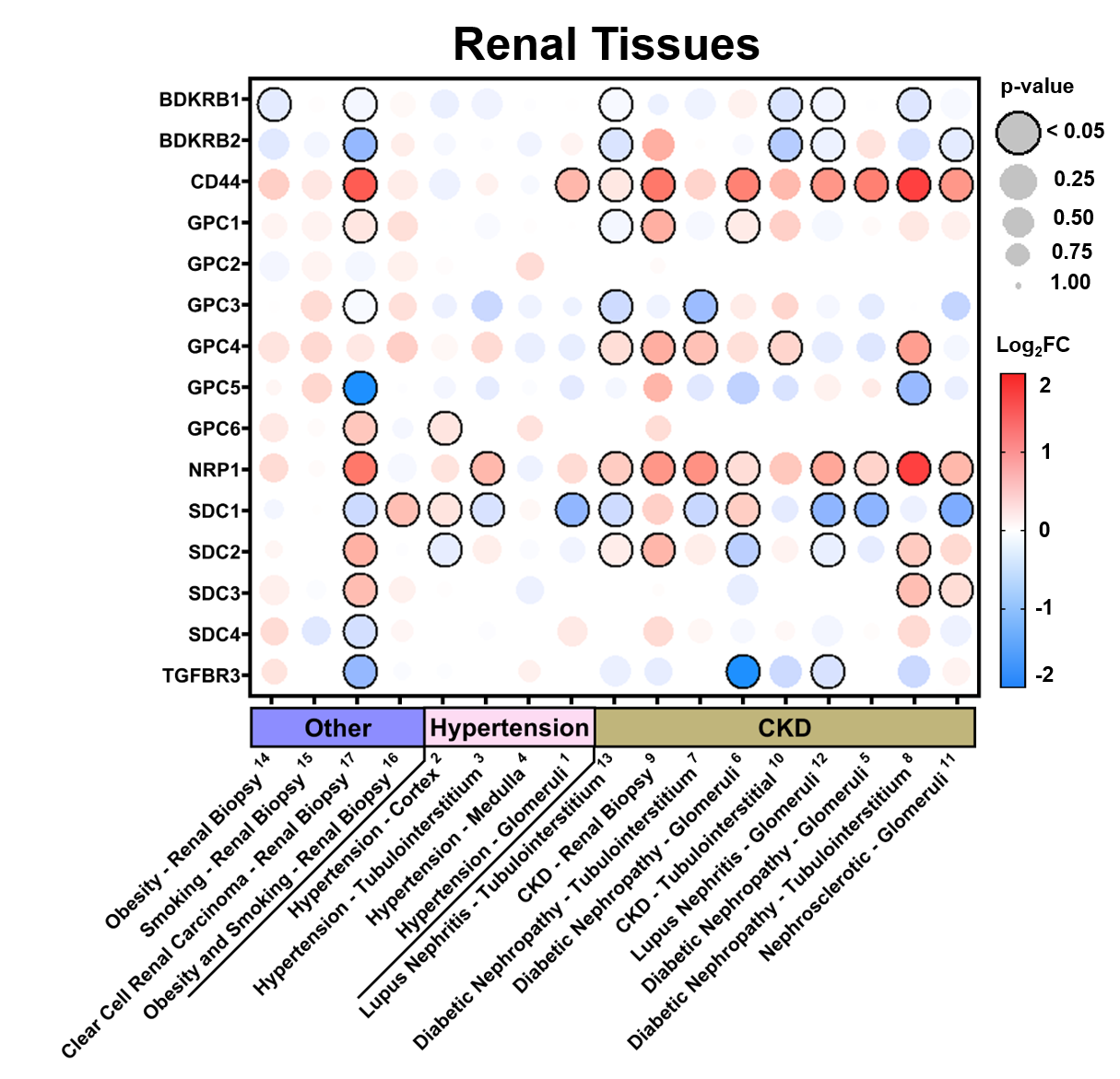


**Figure S3. SARS-CoV-2 viral-entry gene expression in renal tissues across comorbidities.** Expression of select SARS-CoV-2 viral-entry genes in renal tissues across 17 DataSets representing various common comorbidities. Values are displayed as logarithm to base 2 of the fold change (log2FC) in comparison with respective unaffected control samples from each DataSet. Comorbidity groups and each DataSet within are sorted from greatest (left) to least (right) expression of ACE2. Heatmap coloration is set to a scale of -2 (blue) to 2 (red) and values beyond this range are shown as either -2 or 2, respectively. Statistical significance is represented proportional to dot size where largest dot size indicates highest statistical significance as indicated in the key and a black border designates p < 0.05. Superscript numbers link each DataSet to additional information found in Appendix A and Supplementary File 1. CKD = chronic kidney disease.


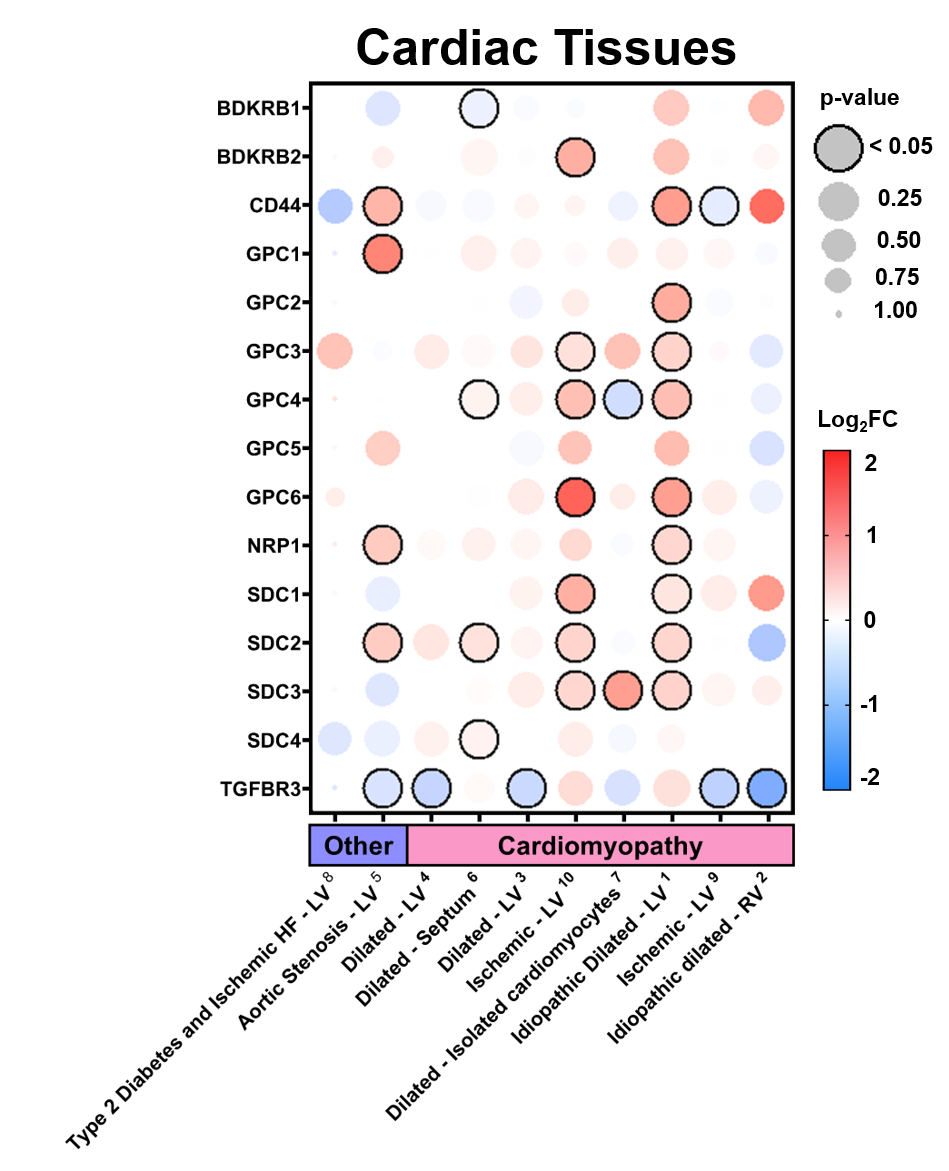


**Figure S4. SARS-CoV-2 viral-entry gene expression in cardiac tissues across comorbidities.** Expression of select SARS-CoV-2 viral-entry genes in cardiac tissues across 10 DataSets representing various common comorbidities. Values are displayed as logarithm to base 2 of the fold change (log2FC) in comparison with respective unaffected control samples from each DataSet. Comorbidity groups and each DataSet within are sorted from greatest (left) to least (right) expression of ACE2. Heatmap coloration is set to a scale of -2 (blue) to 2 (red) and values beyond this range are shown as either -2 or 2, respectively. Statistical significance is represented proportional to dot size where largest dot size indicates highest statistical significance as indicated in the key and a black border designates p < 0.05. Superscript numbers link each DataSet to additional information found in Appendix A and Supplementary File 1. HF = heart failure; LV = left ventricle; RV = right ventricle.


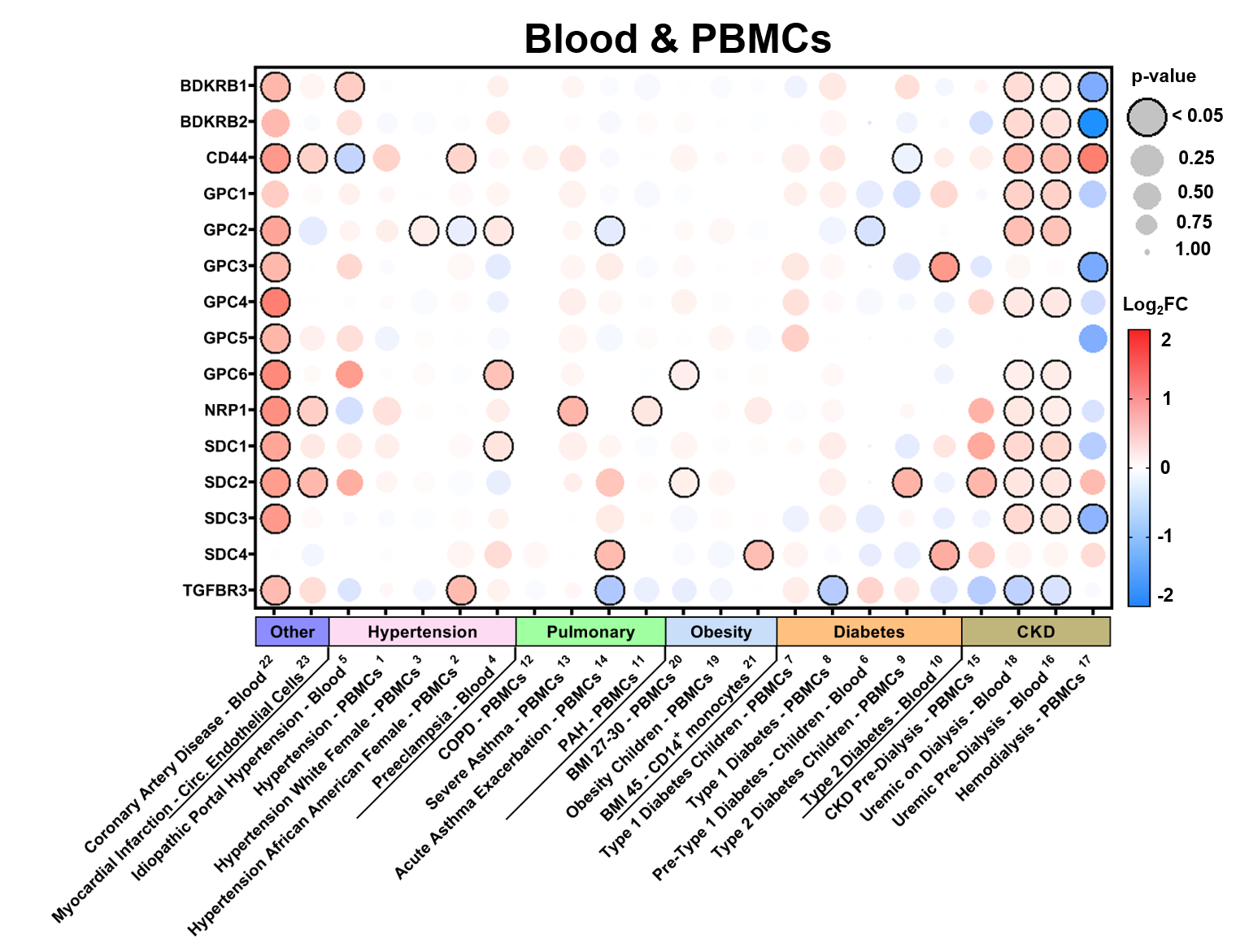


**Figure S5. SARS-CoV-2 viral-entry gene expression in blood tissues across comorbidities.** Expression of select SARS-CoV-2 viral-entry genes in blood tissues across 23 DataSets representing various common comorbidities. Values are displayed as logarithm to base 2 of the fold change (log2FC) in comparison with respective unaffected control samples from each DataSet. Comorbidity groups and each DataSet within are sorted from greatest (left) to least (right) expression of ACE2. Heatmap coloration is set to a scale of -2 (blue) to 2 (red) and values beyond this range are shown as either -2 or 2, respectively. Statistical significance is represented proportional to dot size where largest dot size indicates highest statistical significance as indicated in the key and a black border designates p < 0.05. Superscript numbers link each DataSet to additional information found in Appendix A and Supplementary File 1. BMI = body mass index; CKD = chronic kidney disease; COPD = chronic obstructive pulmonary disease; PBMCs = peripheral blood mononuclear cells; PAH = pulmonary arterial hypertension.
