# Supplementary figures and images for: Impact of Comorbidities on SARS-CoV-2 Viral Entry-Related Genes
Source: J Pers Med. 2020 Sep 25;10(4):146. doi: 10.3390/jpm10040146 (PMC7720121; doi:10.3390/jpm10040146)

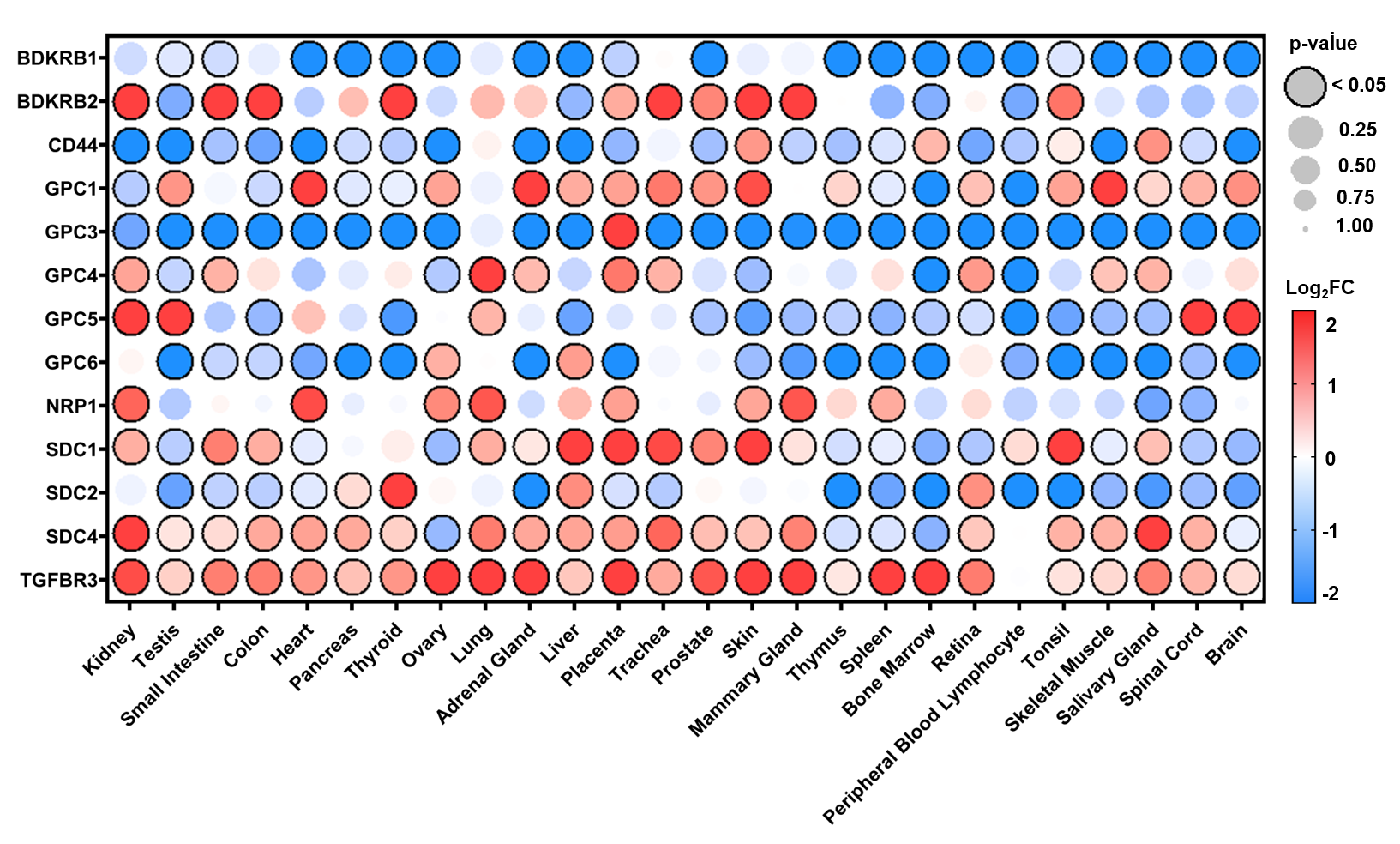

Supplement: Supplementary file 1 [file jpm-10-00146-s001.zip › Figure S1.tif]

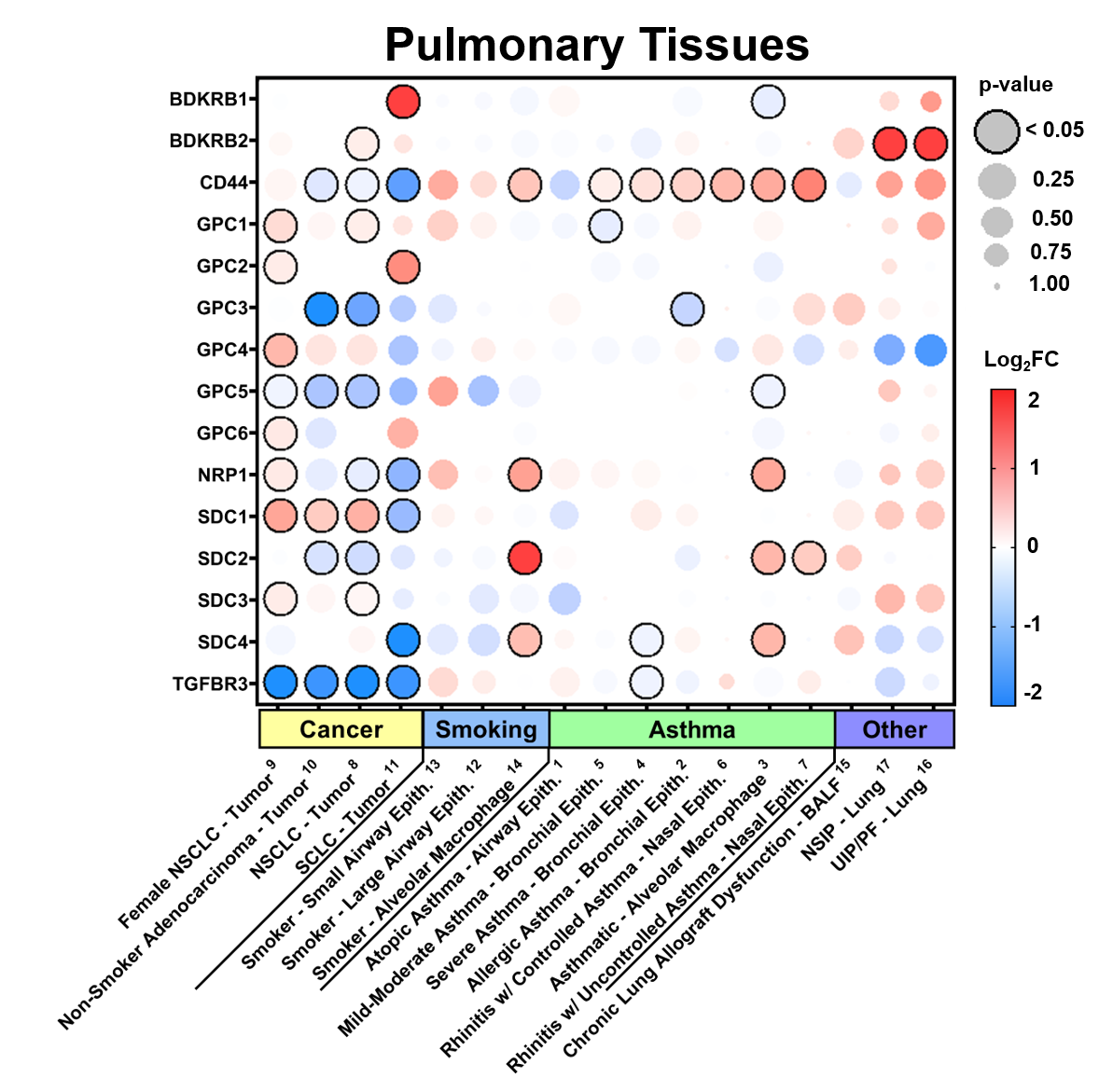

Supplement: Supplementary file 1 [file jpm-10-00146-s001.zip › Figure S2.tif]

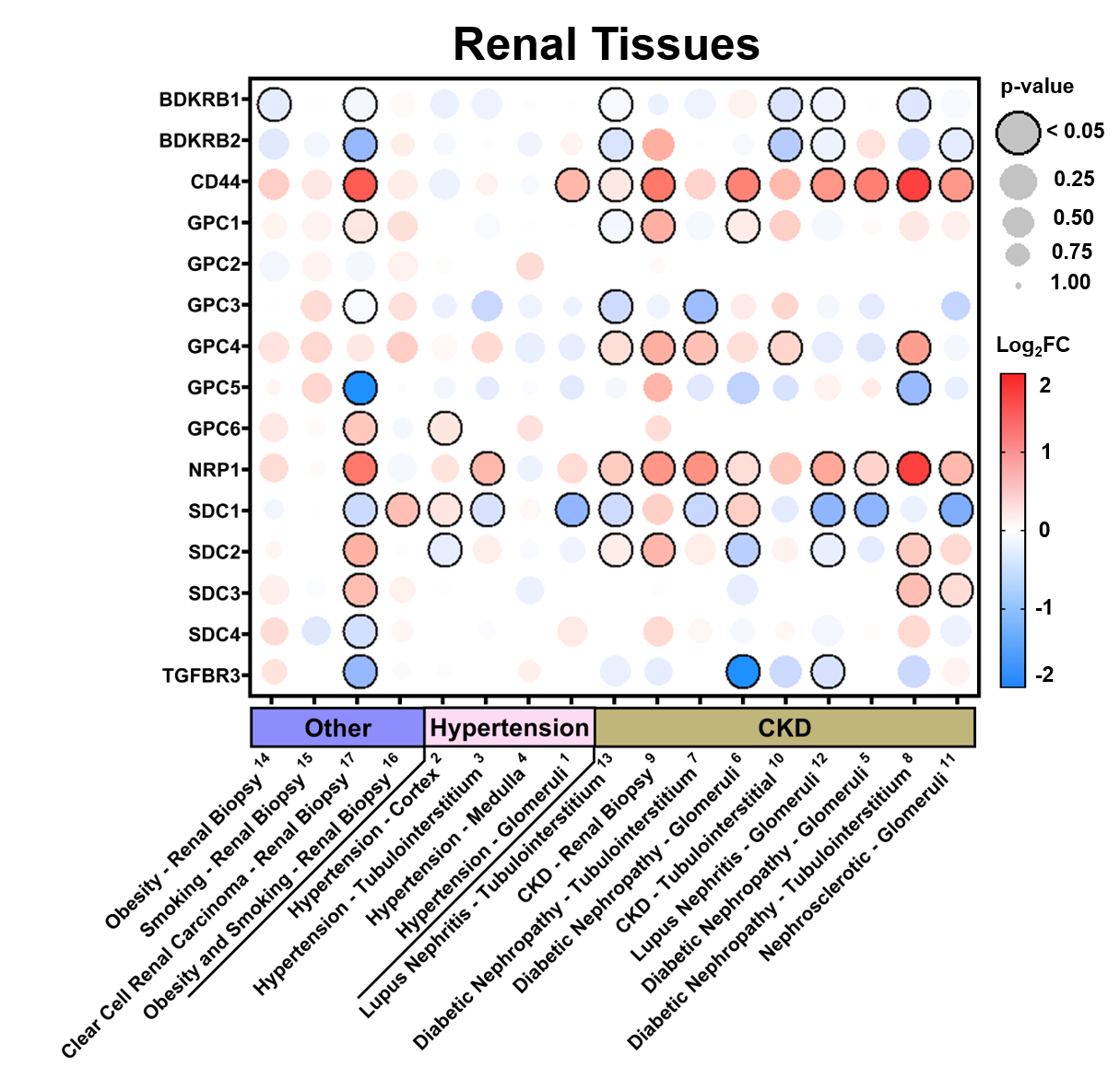

Supplement: Supplementary file 1 [file jpm-10-00146-s001.zip › Figure S3.tif]

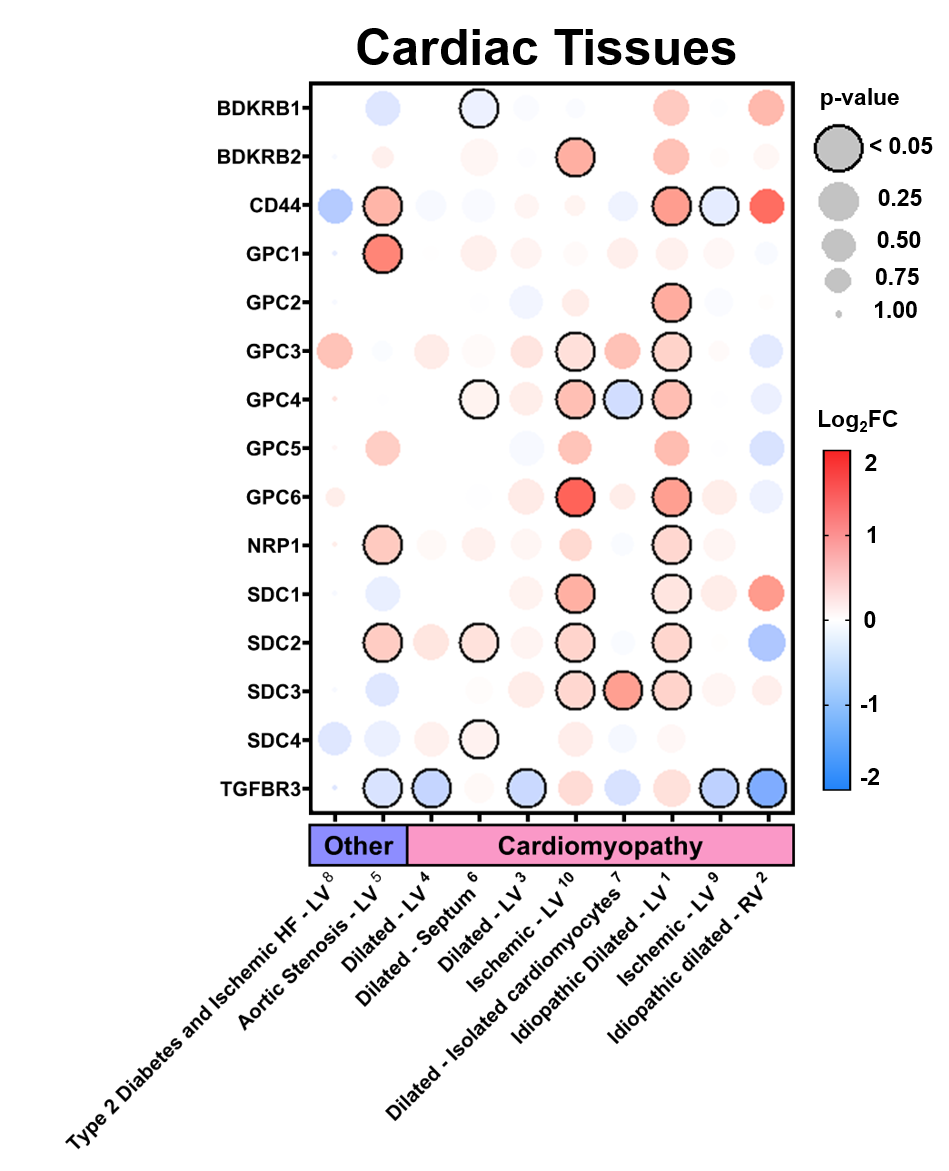

Supplement: Supplementary file 1 [file jpm-10-00146-s001.zip › Figure S4.tif]

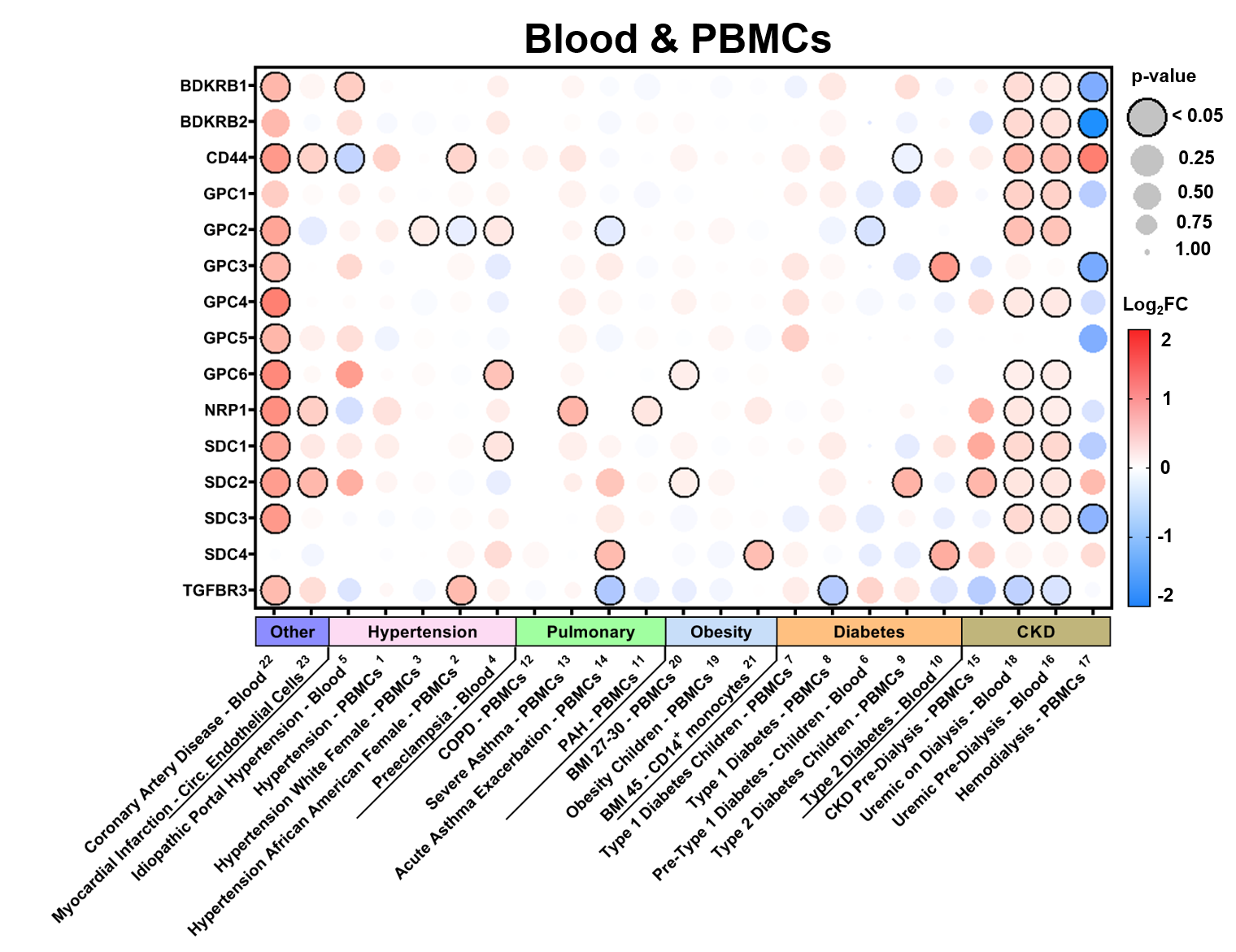

Supplement: Supplementary file 1 [file jpm-10-00146-s001.zip › Figure S5.tif]
